# Effects of *Lactiplantibacillus plantarum* OLL2712 on Memory Function in Older Adults with Declining Memory: A Randomized Placebo-Controlled Trial

**DOI:** 10.3390/nu14204300

**Published:** 2022-10-14

**Authors:** Keisuke Sakurai, Takayuki Toshimitsu, Erika Okada, Saya Anzai, Izumi Shiraishi, Noriko Inamura, Satoru Kobayashi, Toshihiro Sashihara, Tatsuhiro Hisatsune

**Affiliations:** 1 Department of Integrated Biosciences, Graduate School of Frontier Sciences, The University of Tokyo, Kashiwa 277-8562, Japan; 2 Food Microbiology Research Laboratories, Applied Microbiology Research Department, Division of Research and Development, Meiji Co., Ltd., Hachiouji 192-0919, Japan; 3 Urban Design Center Kashiwanoha (UDCK), Kashiwa 277-0871, Japan; 4 Community Health Promotion Laboratory, Mitsui Fudosan, Co., Ltd., Kashiwa 277-8519, Japan

**Keywords:** *Lactiplantibacillus*, probiotics, memory, mild cognitive impairment, aging, gut microbiota

## Abstract

The use of probiotics is expected to be an intervention in neurodegenerative conditions that cause dementia owing to their ability to modulate neuroinflammatory responses via the microbiome-gut–brain axis. Therefore, we selected *Lactiplantibacillus plantarum* OLL2712 (OLL2712), the optimal anti-inflammatory lactic acid bacteria strain with high IL-10-inducing activity in immune cells, and aimed to verify its protective effects on memory function in older adults. A 12-week, randomized, double-blind, placebo-controlled trial was performed with older adults over the age of 65 years with declining memory. The participants consumed either powder containing heat-treated OLL2712 cells or placebo. Memory function was assessed using a computer-assisted cognitive test, Cognitrax. Daily dietary nutrient intake was assessed using the Brief-type Self-administered Diet History Questionnaire (BDHQ). The composition of the gut microbiota was analyzed by fecal DNA extraction and 16S rDNA sequencing. Data from 78 participants who completed the entire procedure were analyzed, and significant improvements in composite memory and visual memory scores were observed in the active group, after accounting for the effect of daily nutritional intake (*p* = 0.044 and *p* = 0.021, respectively). In addition, the active group had a lower abundance ratio of Lachnoclostridium, Monoglobus, and Oscillibacter genera, which have been reported to be involved in inflammation. The present study suggests that OLL2712 ingestion has protective effects against memory function decline in older adults.

## 1. Introduction

According to a WHO report on dementia in 2021, more than 55 million people worldwide experience dementia, with nearly 10 million new cases each year [1]. As the global population ages, the number of patients increases, and it is estimated that the number of patients with dementia will exceed 150 million by 2050 [2]. The increase in the number of patients with dementia is not only a problem for the patients themselves, but also a serious issue from an economic and social perspective due to the cost of medical care and the psychological burden on caregivers [3,4]. On the Alzheimer’s disease (AD) spectrum, which is the most common cause of dementia, the typical course of the disease is onset by impaired memory. As the disease progresses, other cognitive domains, such as language, visuospatial cognition, and executive function, are impaired, gradually making it impossible to maintain independence in daily life [5]. In particular, the preclinical stage, in which daily functions and behaviors are not significantly affected, is classified as mild cognitive impairment (MCI), and the typical symptom is memory impairment. More than 90% of amnestic-MCI is associated with AD pathology [6]. Currently, there is no treatment to stop or reverse the progression of AD; however, longitudinal studies have shown that it is possible to regress to a state of cognitive normality (CN) in the MCI stage [7,8]. Therefore, to reduce the number of patients with dementia, it is important to provide appropriate interventions to prevent cognitive decline during the non-dementia stages such as CN and MCI. However, there is no evidence to support the use of pharmacotherapy to protect cognitive function in patients with CN or MCI [9]. Conversely, since certain lifestyle factors, such as exercise, smoking, diabetes, obesity, and diet, are known to affect the risk of developing AD, lifestyle interventions in the preclinical stage are considered one of the few options for controlling the development of AD [10,11]. Our previous randomized controlled trial (RCT) studies have shown that a nutritional intervention approach with anserine, carnosine, or tea leaf extract for older adults with CN and patients with MCI maintains cognitive function [12,13]. 

The central nervous system (CNS) and gastrointestinal tract form a bidirectional network called the brain–gut axis, which is mediated by systems such as the nervous system, immune system, and endocrine system. In recent years, the microbiota has become increasingly important as a regulator of the brain–gut axis, and this concept has been extended to the microbiota-gut–brain axis [14]. Several animal models and human studies have shown that the gut microbiota is closely associated with the development of AD [15], and a microbiota-gut–brain axis-mediated dietary intervention approach through modulation of the gut microbiota and metabolites by probiotic/prebiotic intake is considered a new treatment candidate for AD [16,17,18]. In addition, meta-analysis has suggested that probiotic supplementation may improve cognitive function, especially in patients with MCI [19].

Although AD is a neurodegenerative disease characterized by extracellular amyloid-β (Aβ) plaques and intra-neuronal deposition of neurofibrillary tangles (NFTs), recent studies suggest that neuroinflammation is closely associated with the pathogenesis of AD [20]. Neuroinflammation refers to the inflammatory response in the CNS associated with neuronal damage [21], and the major players in neuroinflammation are microglia, astrocytes, the complement system, and cytokines; with cytokines in particular playing a central role in neuroinflammation in AD [22]. Inflammatory cytokines produced in both the brain and peripheral nervous system can cause pathological cell death [23], and levels of inflammatory cytokines, such as interleukin (IL)-1β and tumor necrosis factor (TNF), have been shown to be abnormally elevated in patients with AD [24].

IL-10 is a potent anti-inflammatory cytokine that is expressed in microglia, astrocytes, and oligodendrocytes in the brain and plays an important role in limiting neuroinflammatory processes by inhibiting the production of inflammatory cytokines, such as TNF-α, IL-1β, IL-6, and interferon-γ, secretion [25,26]. In addition to its anti-inflammatory effects, IL-10 also plays a neuroprotective role in the CNS by promoting anti-apoptosis and cell survival [27]. *Lactiplantibacillus plantarum* OLL2712 (OLL2712) has been identified as a lactic acid bacteria (LAB) strain with the highest level of inducibility of IL-10 production of Meiji Co., Ltd. LAB libraries and has been shown to suppress chronic inflammation and improve metabolic disorders in in vitro and in vivo models [28]. In particular, it has been reported that the induction activity of IL-10 production on immune cells is significantly higher in heat-treated OLL2712 cells than in alive cells [29]. RCTs of OLL2712 intake in humans have also reported beneficial effects on glucose metabolism and chronic inflammation in healthy adults with prediabetes [30], as well as on abdominal fat accumulation and chronic inflammation in overweight healthy adults [31].

Thus, what distinguishes OLL2712 from other probiotics is its high anti-inflammatory activity due to its higher ability to induce IL-10 production, and it is hypothesized that OLL2712 acts against neuroinflammation through the microbiota-gut-brain axis, thereby exerting a protective effect against cognitive decline, a symptom of neurodegenerative diseases such as AD. However, the effects of OLL2712 intake on cognitive function have not been studied. Therefore, in the present study, we conducted a randomized, double-blind, placebo-controlled trial designed to test the protective effects of 12 weeks of supplementation with heat-treated OLL2712 cells on memory function in older adults.

## 2. Materials and Methods

### 2.1. Study Design and Participants

**Figure 1:**
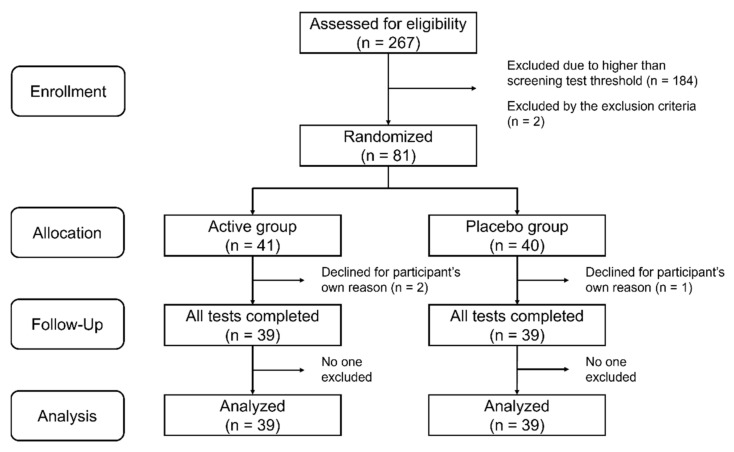
Flow chart showing the number of participants in the active and placebo groups at each stage of the study.

We performed a double-blind placebo-controlled trial randomizing 1:1 Active:Placebo at a single site in Kashiwa City, Chiba, Japan between October 2020 and March 2021 (for recruitment and follow-up). The trial was conducted according to the guidelines of the Declaration of Helsinki and approved by the Ethics Committee of the University of Tokyo (ethical approval code 21-273 and date of approval 25 October 2021). This trial was registered with the UMIN Clinical Trials Registry (ID: UMIN000046179). Written informed consent was obtained from all participants prior to screening. The process by which the participants underwent the screening and the subsequent data analysis is shown in Figure 1. This article conforms to the CONsolidated Standards of Reporting Trials (CONSORT) 2010 guideline (Supplementary Materials Table S1).

We recruited adults over the age of 65 years living in the surrounding areas of Kashiwa City from our volunteer database as screen test participants. In the screening test, the Japanese version of the word list memory test (MCI Screen) [32] was used to identify those with early memory deterioration. In the MCI Screen, a scoring algorithm calculates the Memory Performance Index (MPI) score based on patient test results, age, education, and race [33]. The MPI score is quantified on a scale from 0 to 100 and can distinguish healthy individuals from those with MCI with 96–97% accuracy [34]. Individuals with an MPI score of less than 60 were candidates for the intervention trial. Individuals with serious systemic illnesses, diagnosed with dementia, or allergic to dairy products were excluded from the screening test. The sample size was calculated to be 36 per group, assuming a significance level of 0.05 for a two-tailed test, a power of 80%, a standard deviation of 9 for the intervention change in the composite memory score of Cognitrax for both groups, and a detection of 6 points for the difference. Participants were randomly allocated to the active or placebo group in an approximate 1:1 ratio. The actual allocation was made by Imepro Inc. (Tokyo, Japan) in a manner that matched the age and sex ratios of the two groups. A total of 81 participants underwent a start-up test prior to the intervention. They then received OLL2712 or placebo once daily for 12 weeks. Three participants dropped out during the course of the study, and 78 participants completed the follow-up test. Data from these 78 participants were included in the analysis. The number of participants used in the analysis met the required sample size. There were no reported harms or unintended effects in each group.

### 2.2. Test Foods

In this study, heat-treated OLL2712 cells were used instead of alive cells due to their higher activity in inducing IL-10 production [29]. OLL2712 was cultured in a medium containing enzymatic degradation products of skimmed milk powder until late in the logarithmic growth phase. The culture was concentrated 25-fold and heated to 60 °C for 10 min. The concentrated heated bacterial solution was spray-dried to powder and mixed with TK-16AG dextrin (Matsutani Chemical Industry Co., Ltd., Hyogo, Japan) at an addition rate of 3.9% until completely homogeneous. The number of OLL2712 cells in the 1 g group was greater than 5 × 10^9^. The safety of OLL2712 at the same dose (5 × 10^9^ or more) for 12 weeks as in this study has been demonstrated in three previous clinical studies [30,31,35]. The mixed powder was packed in sticks of 1 g/package, and one packet was used daily as the test food. In contrast, the placebo group was administered a stick filled with 1 g of TK-16AG dextrin without OLL2712. Each test food sample was then stored at room temperature (15–25 °C).

### 2.3. Memory Functions Tests

The participants’ memory function was measured using the visual memory test (VIM) and verbal memory test (VBM), the memory domains of Cognitrax, which was developed as the Japanese version of the CNS Vital Signs (CNSVS) [33]. In the two memory tests, the participants memorized 15 geometric shapes (VIM) or words (VBM). VIM and VBM testing procedures and score calculation methods are shown in Supplementary Table S2.The two memory tests were conducted at baseline (0 week) and follow-up (12 weeks). The composite memory score, the sum of the two memory scores, was also included in the evaluation. Higher scores indicated better memory function.

### 2.4. Assessment of Daily Nutritional Intake

Dietary questionnaires are useful way to assess daily nutritional intake. However, since food habits differ from country to country, it is necessary to use questionnaires optimized for each country’s food culture in order to obtain reliable results [36]. In this study, nutrient intake in the daily diet was assessed using the Brief Self-administered Diet History Questionnaire (BDHQ) [37]. The BDHQ is a questionnaire designed to estimate nutrient and food intake, optimized for the Japanese diet, and consists of 80 questions to calculate the estimated intake of 58 foods and over 100 nutrients. It has been validated by comparison with semi-weighed dietary records [38]. A sample of the BDHQ used in this study can be referred to at the following URL (http://www.nutrepi.m.u-tokyo.ac.jp/dhq/BDHQ1-1.pdf (accessed on 11 September 2022)). BDHQ estimates of daily nutrient intakes do not take into account information on nutritional supplements due to the low use of such products in Japan [37]. Therefore, all estimated daily nutrient intakes such as cholesterol and vitamins calculated in this study are derived from regular foods. This questionnaire was administered at baseline.

### 2.5. Gut Microbiota Analysis

Fecal samples were collected by the participants on any day during the week prior to the follow-up test. The samples were homogenized using FastPrep-24 5G (MP Biomedicals, Irvine, CA, USA) with 0.1 mm zirconia beads (EZ-Extract for DNA/RNA, AMR, Tokyo, Japan). DNA was extracted from the fecal samples using the Maxwell RSC PureFood GMO and Authentication Kit (Promega Corporation, Madison, WI, USA). The V3-V4 region of the 16S ribosomal RNA (rRNA) gene was amplified using PCR with universal bacterial primer sets (5′-TCGTCGGCAGCGTCAGATGTGTATAAGAGACAGCCTACGGGNGGCWGCAG-3′ and 5′-GTCTCGTGGGCTCGGAGATGTGTATAAGAGACAGGACTACHVGGGTATCTAATCC-3′) and sequenced using the MiSeq Reagent kit v3 (600 cycles) (Illumina Inc., San Diego, CA, USA). The sequence data were analyzed using QIIME2 (https://qiime2.org (accessed on 11 September 2022) to calculate the ratio of each genus and alpha diversity was expressed using the Shannon index and Simpson index. In addition, the relative composition of the gut microbiota at the genus level was compared between the groups. When comparing the groups, only the genera with the top 20% relative abundance in the gut microbiota were included in the analysis.

### 2.6. Statistical Analysis

All data analyses were performed using Bell Curve for Excel ver. 4.01 (Social Survey Research Information Co. Ltd., Tokyo, Japan). Statistical significance was set at *p* < 0.05. The analysis of the effects of OLL2712 on memory function without the influence of daily nutritional intake and participant characteristics was performed using two-way time × group analysis to test the effects of OLL2712 on memory function. In addition, to analyze the effects of OLL2712 on memory function, taking into account the influence of daily nutritional intake and participant characteristics, multiple regression analysis was performed using the major nutrients in the six macronutrients (protein, carbohydrate, mineral, saturated fatty acids, monounsaturated fatty acids, polyunsaturated fatty acids, cholesterol, water-soluble vitamins, fat-soluble vitamins, dietary fiber), covariates (age, sex, years of education, body mass index (BMI)) as candidate explanatory variables, changes in scores for each memory function domain in Cognitrax as the objective variable, and group as the fixed factor. As our previous studies have shown that age, sex, and years of education can affect cognitive test scores in older adults [39], and BMI has also been correlated in some studies [40], we adopted these as candidate explanatory variables. In addition, because our previous study on the relationship between cognitive function and dietary habits in older adults showed that monounsaturated fatty acids and fat-soluble vitamins were related to cognitive function scores [13,41], we divided the data on lipids and vitamins into monounsaturated fatty acids, polyunsaturated fatty acids, cholesterol, water-soluble vitamins, and fat-soluble vitamins as candidate explanatory variables. Mineral intake was the sum of sodium, potassium, calcium, magnesium, phosphorus, iron, zinc, copper, and manganese intakes standardized by the Z-score. Water-soluble vitamin intake was the sum of vitamin B1, vitamin B2, niacin, vitamin B6, folic acid, vitamin B12, biotin, pantothenic acid, and vitamin C intakes, standardized by the Z-score. Fat-soluble vitamin intake was the sum of vitamin A, vitamin D, vitamin E, and vitamin K intakes standardized by the Z-score. In the multiple regression analysis, all variables were standardized using the Z-score. If the significance was *p* ≤ 0.20 for the primary outcome, the explanatory variables were included in a stepwise multiple regression analysis. If the explanatory variables remained statistically significant at the *p* ≤ 0.20 level, they were considered candidates for variables in the final multiple regression model, and the combination of the variables with the lowest Akaike information criterion was adopted as the final multiple regression model. According to Hair et al., multicollinearity becomes a problem when the variance inflation factor (VIF) exceeds 10 [42]; however, since there were no factors with VIF exceeding 10 in this study, we determined that there was no multicollinearity problem among the explanatory variables. In the multiple regression analysis with composite memory change as the objective variable, since fat-soluble vitamin intake was incorporated into the final regression equation as a significant predictor in the primary multiple regression analysis, it was split again into vitamins A, D, E, and K intake; additional multiple regression analysis was conducted with the covariate and cholesterol intake, which was also a significant predictor, as candidate explanatory variables to identify fat-soluble vitamins that influence composite memory performance concurrent with OLL2712 intake. The Mann-Whitney *U* test was used for intergroup comparisons in the analysis of the gut microbiota.

## 3. Results

### 3.1. Participant Characteristics

Of the 81 participants, 78 completed all trials and their data were used in the analysis. The characteristics of the participants used in this analysis are listed in Table 1. The active and placebo groups used in the analysis included 39 participants each, with no significant differences in age, sex, years of education, or BMI between the groups (Table 1).

### 3.2. Analysis of the Effects of OLL2712 on Memory Function without the Influence of Daily Nutritional Intake and Participant Characteristics

Table 2 shows the pre- and post-intervention changes in scores for each Cognitrax memory function domain. In the visual memory domain, the active group showed a significant improvement (time × group interaction, *p* = 0.044; Table 2). No significant difference was found in the composite memory domain; however, the active group showed an improvement tendency (time × group interaction, *p* = 0.058; Table 2). In the verbal memory domain, there were no significant differences in the score changes between the groups (time × group interaction, *p* = 0.359; Table 2).

### 3.3. Analysis of the Effects of OLL2712 on Memory Function with the Influence of Daily Nutritional Intake and Participant Characteristics

To conduct an analysis that considers the characteristics of the participants and the influence of their daily diet on the effects of OLL2712 intake, a multiple regression analysis was performed, with covariates and intake of major nutrients based on the BDHQ as candidate explanatory variables, each memory function domain of Cognitrax as an explanatory variable, and group as a fixed factor. Multiple regression analysis with the change in visual memory score as the objective variable resulted in a significant regression equation (*P* = 0.034, *R*^2^*_adj_* = 0.061; Table 3). Group and daily dietary cholesterol intakes were incorporated into the final regression equation model, which showed that OLL2712 intake (belonging to the active group) had a significant positive effect on changes in visual memory scores (*p* = 0.021; Table 3). Multiple regression analysis with change in the composite memory score as the objective variable resulted in a significant regression equation (*P* = 0.005, *R*^2^*_adj_* = 0.125; Table 4). The group and daily dietary cholesterol and fat-soluble vitamin intakes were incorporated into the final regression equation model. The intake of OLL2712 had a significant positive effect on the change in the composite memory score (*p* = 0.044; Table 4). Cholesterol intake had a significant negative effect on changes in the composite memory score (*p* = 0.006; Table 4), whereas fat-soluble vitamin intake had a significant positive effect (*p* = 0.006; Table 4). Subsequently, fat-soluble vitamin intake, which was a significant predictor in the multiple regression analysis of the composite memory score, was divided into the intake of vitamins A, D, E, and K, and an additional multiple regression analysis was performed with the covariates and cholesterol intake as candidate explanatory variables. Only vitamin K was incorporated into the final regression equation as a significant predictor, along with group and cholesterol levels (*P* = 0.039, *R*^2^*_adj_* = 0.088; Table 5). The intakes of OLL2712 (belonging to the active group) and vitamin K had a significant positive effect on the change in the composite memory score (*p* = 0.021 and *p* = 0.043, respectively; Table 5), and cholesterol intake had a significant negative effect (*p* = 0.018; Table 5). In addition, sex and years of education were incorporated into the final regression equation from the covariates but were not significant predictors. However, multiple regression analysis with changes in verbal memory score as the objective variable did not yield a significant regression equation.

### 3.4. Gut Microbiota Analysis

**Figure 2:**
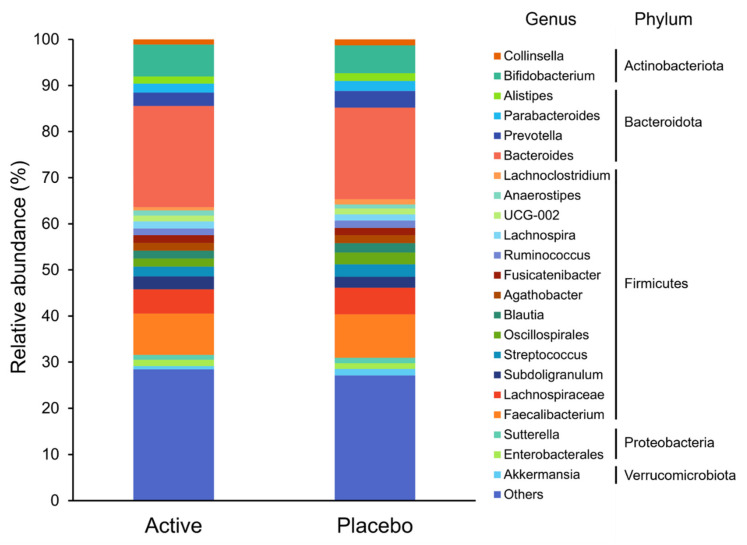
The composition of the gut microbiota at the genus level. Taxa with low relative abundances of <1% are included in others.

**Figure 3:**
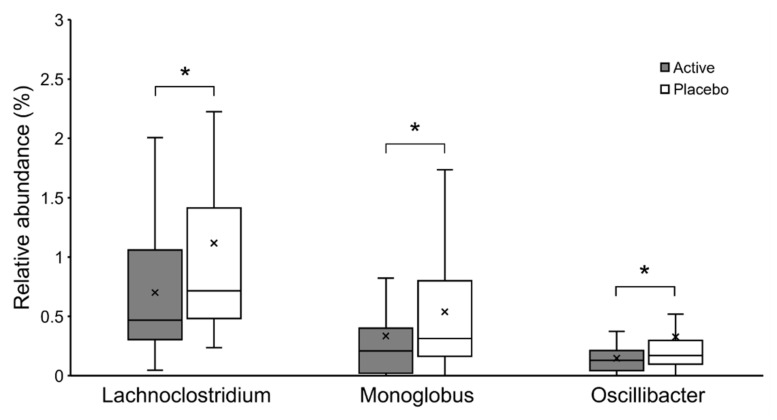
The relative abundance of Lachnoclostridium, Monoglobus, and Oscillibacter shown in Box-and-whisker diagram. * *p* < 0.05.

Based on the Shannon and Simpson indices, there were no significant differences in the alpha diversity of the gut microbiota between the groups (*p* = 0.708 and *p* = 0.853, respectively). The composition of the gut microbiota at the genus level is shown in Figure 2. Genus-level analysis showed that the relative abundance ratios of Lachnoclostridium, Monoglobus, and Oscillibacter were significantly lower in the active group (*p* = 0.033, *p* = 0.015, and *p* = 0.016, respectively; Figure 3).

## 4. Discussion

In this study, a randomized, double-blind, placebo-controlled trial of 12 weeks of supplementation with heat-treated OLL2712 cells was conducted to determine the effects of anti-inflammatory LAB supplementation on memory function in older adults with early memory decline. The results of the analysis, which considered the effects of daily nutritional intake and participant characteristics, showed that OLL2712 consumption had a protective effect on memory function in older adults. This was shown in the between-group difference in the score change in the composite and visual memory domains of Cognitrax from start-up to follow-up by multiple regression analysis. A simple analysis of the Cognitrax score changes also showed a positive effect of OLL2712 cell ingestion on visual memory. However, there was no significant effect of OLL2712 intake on verbal memory in either of the analyses. Since Cognitrax calculates the composite memory score as the sum of visual and verbal memory scores [33], it appears that the protective effect of OLL2712 on memory function is particularly affected by improvements in visual memory. Previous studies on pre-MCI, an earlier stage of MCI, have reported that patients with pre-MCI with lower visual memory scores, but not verbal memory, have a higher rate of progression to the MCI stage [43]. In other words, visual memory loss is a predictor of MCI progression and the earliest symptom of the AD spectrum, as it appears before the onset of clinical MCI. Recalling a list of shapes has a higher error rate than recalling a list of words in Cognitrax, suggesting that this is a more difficult task [33]. Therefore, we considered that a significant intervention effect of OLL2712 intake was obtained in visual memory, a task of high difficulty, and a symptom that shows changes from the beginning of the AD spectrum.

In the gut microbiota analysis, the bacterial composition of the active group showed significantly lower abundance ratios of Lachnoclostridium, Monoglobus, and Oscillibacter. Lachnoclostridium and Oscillibacter are known to increase in abundance with increased inflammation due to carcinogenesis induction, and decrease in abundance with suppression of inflammation by administration of probiotics in a mouse model [44,45]. It is also known that Lachnoclostridium and Oscillibacter are significantly increased in a mouse model fed a high-fat diet [46], and administration of fermentable dietary fiber has been shown to decrease their abundance ratio and improve the inflammatory response [47]. Oscillibacter has also been reported to be an opportunistic pathogen in the human gut microbiota [48], and its increase correlates with abnormal intestinal permeability [49]. Monoglobus is known to act as a pectinolytic bacterium in the human colon [50], and it has been speculated that the amount of Monoglobus present is positively correlated with blood ammonia levels [51]. Elevated ammonia levels disrupt the barrier function of intestinal epithelial cells, abnormally increase intestinal permeability, and cause a systemic inflammatory response [52]. Since these genera are related to the inflammatory response, these three bacteria may be identified as bacterial biomarkers of inflammatory improvement with the ingestion of OLL2712.

OLL2712 is a LAB with high inducibility for IL-10 production. As IL-10 protects cells from neuroinflammation by suppressing the production of proinflammatory mediators and promoting wound repair signaling [53,54], increasing IL-10 production is considered one of the most effective ways to reduce the harmful effects of excessive neuroinflammation [55]. Neuroinflammation, in addition to Aβ and NFTs, is thought to be one of the underlying mechanisms in the pathogenesis of AD, and several epidemiological studies have suggested that the use of anti-inflammatory agents may reduce the risk of AD [56]. Furthermore, it has been reported that IL-10 expression in the brains of AD model mice enhances neurogenesis and cognitive function [57]. OLL2712 has been consistently found to have an inhibitory effect on systemic chronic inflammation in mouse models and human RCT studies [28,29,30,31,35]. For the intestinal immune system, it has been suggested that OLL2712 act on dendritic cells and macrophages to induce IL-10 production and suppress intestinal inflammation [58]. In the present study, we observed a decrease in the relative abundance of several genera involved in inflammation. Based on these findings, the reduction in these genera is presumably due to the suppression of chronic inflammation via the induction of IL-10 production in the intestinal immune system by OLL2712. It is hypothesized that the administration of OLL2712 suppresses intestinal inflammation and neuroinflammation through the brain–gut axis, resulting in improved memory function.

The intervention effect of OLL2712 intake on composite memory was smaller for those with a higher cholesterol intake in their daily diet. In vivo studies have shown that administration of prebiotics or probiotics is effective in reducing serum or plasma total cholesterol, low-density lipoprotein (LDL)-cholesterol, and triglycerides, or increasing high-density lipoprotein (HDL)-cholesterol [59]; administration of OLL2712 has been reported to significantly increase HDL-cholesterol and improve lipid profiles [31]. The involvement of cholesterol in AD is also supported by studies of the apolipoprotein E (APOE) gene [60], which is involved in lipid metabolism in the brain. Meta-analyses have reported that higher total cholesterol levels in middle age increase the risk of cognitive decline in late life [61]. Furthermore, meta-analyses have also demonstrated a relationship between increased dietary cholesterol intake and increased total cholesterol levels [62]. In light of these findings, it is possible that in this study, high cholesterol intake may have offset the effect of OLL2712 on improving the lipid profile, resulting in no improvement in memory function in individuals with high cholesterol intake.

In contrast, the intervention effect of OLL2712 intake on composite memory was greater in those with a higher intake of fat-soluble vitamins in their daily diet. Fat-soluble vitamins are hydrophobic vitamins that include vitamins A, D, E, and K. The fat-soluble vitamin intake used in the multiple regression analysis in this study was the sum of standardized vitamin A, D, E, and K intakes. There is a strong association between fat-soluble vitamin intake and AD, and fat-soluble vitamins may have an impact on the pathogenesis of AD, both through direct effects on molecular mechanisms and through interference with cellular lipid homeostasis [63]. Additional multiple regression analysis with each fat-soluble vitamin intake as a candidate explanatory variable revealed that only vitamin K was a significant predictor of changes in composite memory performance, concurrent with OLL2712 intake. Vitamin K is a fat-soluble vitamin discovered as a nutrient involved in blood coagulation; however, recent studies have suggested that it has anti-apoptotic and anti-inflammatory effects and may also be involved in neuroinflammation and neurodegeneration through altered sphingolipid profile expression [64]. Cross-sectional studies have reported a positive correlation between vitamin K intake and cognitive function and subjective memory impairment [65,66,67]. The use of vitamin K antagonists may adversely affect cognitive functions, such as visual memory and verbal fluency [68], suggesting that vitamin K intake is associated with cognitive function. Furthermore, in our previous intervention study of older adults, the improvement in cognitive function by supplementation with tea leaf extract, a food rich in vitamin K, was greater in those who consumed less vitamin K in their daily diet [13]. Thus, the effects of fat-soluble vitamin intake, especially vitamin K, on the composite memory changes observed in this study may reflect the additive or synergistic effects of OLL2712 and fat-soluble vitamins on AD pathology, such as neuroinflammation.

Nevertheless, some limitations should be noted. First, this study is a small RCT. The sample size of subjects was calculated assuming a standard deviation of 9 for the intervention change in the composite memory score of Cognitrax for both groups, and a detection of 6 points for the difference. Since the measured standard deviations were generally as expected, even small score differences could be detected as significant improvements if the number of subjects were increased. In fact, the *p*-value for the composite memory score is very close to the significance level (*p* = 0.058), therefore increasing the sample size would clarify the effect of OLL2712 on general memory function. In addition, this study was conducted with healthy elderly volunteers aged over 65 in the same community, which may limit its generalizability to the elderly population. Therefore, future trials will demonstrate a clear improvement in a larger number of cases, although the clinical significance of statistically significant differences must be carefully determined. Second, this study did not quantify metabolites. In particular, short-chain fatty acids (SCFA), a major group of intestinal metabolites produced as end products of anaerobic fermentation by intestinal bacteria, have been reported to have different immunomodulatory effects [69]. To better understand the mechanism of action of OLL2712, we plan to quantify these metabolic organic molecules and identify the main active components in future studies. Although blood samples were not collected in this study, previous intervention studies with OLL2712 in humans have investigated metabolic factors related to glycemic control, lipid profiles, and chronic inflammatory markers using blood samples, and have confirmed the antidiabetic, anti-inflammatory, and lipid profile improving effects of OLL2712 intake [30,31,35]. However, Aβ and tau in the cerebrospinal fluid, fluid markers that reflect senile plaques and NFTs and are two characteristic microstructural features of AD [70], have not been investigated. To clarify the results of this preliminary study and make it more complete, future studies will include additional measurements that include these metabolites and analyze their relationship to the gut microbiota. Third, genetic testing of the participants was not conducted, and the relationship between OLL2712 and genetic polymorphisms related to dementia; such as APOE, related to the risk of developing AD; IL-10, related to immunity; and plasminogen activator inhibitor-1, related to atherosclerosis; remains unknown. Fourth, the analysis of the gut microbiota in this study did not investigate changes in the composition of the gut microbiota, but the composition in a single sample at the time point after the 3-month intervention. We considered the analysis of the amount of change before and after the intervention to be not very effective in the analysis of gut microbiota because of the large intra-subject variability. In order to compare changes in composition before and after the intervention, it may be necessary to reduce variation within the same subject by extracting and standardizing fecal samples from each subject on three or more different days. The finding of a significant difference in genera involved in inflammation after the intervention compared to the placebo in a randomized trial seems to be a meaningful result. However, further studies to confirm their reproducibility are needed by standardization with three-point sampling before and after the intervention to improve the reliability of these genera as bacterial biomarkers of inflammation improvement.

## 5. Conclusions

This is the first RCT to demonstrate the efficacy of OLL2712 in improving memory function in older adults. After 12 weeks of supplementation with OLL2712 in older adults with early memory decline, the OLL2712 intake group showed significant improvement in composite memory and visual memory, and a lower relative abundance of Lachnoclostridium, Monoglobus, and Oscillibacter, the genera involved in inflammation. As there is currently no effective pharmacotherapy established to prevent the onset and progression of cognitive decline in the pre-dementia stage, the results suggest that continuous intake of OLL2712 may be an effective approach to protect memory function in older adults. 

## Figures and Tables

**Table 1:** Participant characteristics.

Item	Active (*n* = 39)	Placebo (*n* = 39)	*p*-Value
Age	76.8 ± 4.6 ^a^	76.9 ± 4.9 ^a^	0.906 ^c^
Sex (Male/Female)	18/21 ^b^	18/21 ^b^	1.000 ^d^
Years of Education	13.9 ± 2.4 ^a^	14.0 ± 2.1 ^a^	0.804 ^c^
BMI	21.7 ± 2.5 ^a^	22.3 ± 3.4 ^a^	0.352 ^c^

The data represent the characteristics of the 78 participants who completed the follow-up tests. ^a^ Mean ± standard deviation. ^b^ Number of people. ^c^ *p*-value was determined using Student’s *t*-test. ^d^ *p*-value was calculated using the chi-square test. BMI: body mass index.

**Table 2:** Scores for each memory function domain in Cognitrax.

Domain	Start-Up		Follow-Up		Δ		
	Active	Placebo	Active	Placebo	Active	Placebo	*p*-Value
Visual memory	41.7 ± 5.9	43.3 ± 4.1	42.7 ± 5.0	41.6 ± 4.6	1.1 ± 6.1	−1.6 ± 5.5	0.044 *
Verbal memory	49.6 ± 6.2	49.5 ± 5.1	49.4 ± 6.1	48.1 ± 5.7	−0.2 ± 5.2	−1.4 ± 6.3	0.359
Composite memory	91.3 ± 9.7	92.8 ± 8.0	92.2 ± 9.3	89.7 ± 8.1	0.8 ± 8.3	−3.1 ± 9.6	0.058

Values represent the mean ± standard deviation. Δ: amount of change from baseline. The *p*-value was determined by two-way time × group analysis of score change. * *p* < 0.05.

**Table 3:** Multiple regression analysis of the change in visual memory score and the intake of major nutrients.

Variable	*β*	SE *β*	*p*	VIF
Group	0.267	0.113	0.021 *	1.042
Cholesterol	−0.187	0.113	0.101	1.042
***P***	0.034 *			
***R* ^2^ *_adj_***	0.062			

The group was coded as 1 = active and −1 = placebo. *β*: standardized partial regression coefficient. SE *β*: standard error of the standardized partial regression coefficient. *p*: *p*-value of statistical significance in the partial regression coefficient. VIF: variance inflation factor. *R*^2^_adj_: adjusted coefficient of determination. *P*: *p*-value of statistical significance in the multiple linear regression equation. * *p* < 0.05.

**Table 4:** Multiple regression analysis of the change in composite memory score and the intake of major nutrients.

Variable	*β*	SE *β*	*p*	VIF
Group	0.224	0.109	0.044 *	1.048
Cholesterol	−0.387	0.137	0.006 **	1.653
Fat-soluble vitamins	0.385	0.137	0.006 **	1.639
***P***	0.005 **			
***R* ^2^ *_adj_***	0.125			

The group was coded as 1 = active and −1 = placebo. *β*: standardized partial regression coefficient. SE *β*: standard error of the standardized partial regression coefficient. *p*: *p*-value of statistical significance in the partial regression coefficient. VIF: variance inflation factor. *R*^2^_adj_: adjusted coefficient of determination. *P*: *p*-value of statistical significance in the multiple linear regression equation. * *p* < 0.05, ** *p* < 0.01.

**Table 5:** Multiple regression analysis of the change in composite memory score and the intake of cholesterol and vitamin K.

Variable	*β*	SE *β*	*p*	VIF
Group	0.263	0.112	0.021 *	1.053
Sex	0.223	0.126	0.081	1.339
Years of education	0.164	0.124	0.189	1.295
Cholesterol	−0.308	0.127	0.018 *	1.357
Vitamin K	0.241	0.117	0.043 *	1.158
***P***	0.039 *			
***R* ^2^ *_adj_***	0.088			

The group was coded as 1 = active and −1 = placebo. Sex was coded as −1.08 = male and 0.93 = female. *β*: standardized partial regression coefficient. SE *β*: standard error of the standardized partial regression coefficient. *p*: *p*-value of statistical significance in the partial regression coefficient. VIF: variance inflation factor. *R*^2^_adj_: adjusted coefficient of determination. *P*: *p*-value of statistical significance in multiple linear regression equation. * *p* < 0.05.

## Data Availability

Data supporting the results of this study are available from the corresponding author upon reasonable request.

## Supplementary Materials

The following supporting information can be downloaded at: https://www.mdpi.com/article/10.3390/nu14204300/s1. Table S1: CONSORT 2010 checklist of information to include when reporting a randomised trial. Table S2: Measures of visual, verbal, and composite memory in Cognitrax.

## References

[1] World Health Organization Dementia. https://www.who.int/news-room/fact-sheets/detail/dementia.

[2] GBD 2019 Dementia Forecasting Collaborators (2022). Estimation of the global prevalence of dementia in 2019 and forecasted prevalence in 2050: An analysis for the Global Burden of Disease Study 2019. Lancet Public Health.

[3] Cantarero-Prieto D., Leon P.L., Blázquez-Fernández C., Juan P.S., Cobo C.M.S. (2020). The economic cost of dementia: A systematic review. Dementia.

[4] Cheng S.-T. (2017). Dementia Caregiver Burden: A Research Update and Critical Analysis. Curr. Psychiatry Rep..

[5] McKhann G.M., Knopman D.S., Chertkow H., Hyman B.T., Jack C.R., Kawas C.H., Klunk W.E., Koroshetz W.J., Manly J.J., Mayeux R. (2011). The diagnosis of dementia due to Alzheimer’s disease: Recommendations from the National Institute on Aging-Alzheimer’s Association workgroups on diagnostic guidelines for Alzheimer’s disease. Alzheimer’s Dement..

[6] Atri A. (2019). The Alzheimer’s Disease Clinical Spectrum: Diagnosis and Management. Med. Clin. North Am..

[7] Ganguli M., Jia Y., Hughes T.F., Snitz B.E., Chang C.-C.H., Berman S.B., Sullivan K., Kamboh M.I. (2019). Mild Cognitive Impairment that Does Not Progress to Dementia: A Population-Based Study. J. Am. Geriatr. Soc..

[8] Shimada H., Makizako H., Park H., Doi T., Lee S. (2017). Validity of the National Center for Geriatrics and Gerontology-Functional Assessment Tool and Mini-Mental State Examination for detecting the incidence of dementia in older Japanese adults. Geriatr. Gerontol. Int..

[9] Fink H.A., Jutkowitz E., McCarten J.R., Hemmy L.S., Butler M., Davila H., Ratner E., Calvert C., Barclay T.R., Brasure M. (2018). Pharmacologic Interventions to Prevent Cognitive Decline, Mild Cognitive Impairment, and Clinical Alzheimer-Type Dementia: A Systematic Review. Ann. Intern. Med..

[10] Kivipelto M., Mangialasche F., Ngandu T. (2018). Lifestyle interventions to prevent cognitive impairment, dementia and Alzheimer disease. Nat. Rev. Neurol..

[11] Yusufov M., Weyandt L.L., Piryatinsky I. (2017). Alzheimer’s disease and diet: A systematic review. Int. J. Neurosci..

[12] Masuoka N., Yoshimine C., Hori M., Tanaka M., Asada T., Abe K., Hisatsune T. (2019). Effects of Anserine/Carnosine Supplementation on Mild Cognitive Impairment with APOE4. Nutrients.

[13] Sakurai K., Shen C., Ezaki Y., Inamura N., Fukushima Y., Masuoka N., Hisatsune T. (2020). Effects of Matcha Green Tea Powder on Cognitive Functions of Community-Dwelling Elderly Individuals. Nutrients.

[14] Cryan J.F., O’Riordan K.J., Cowan C.S.M., Sandhu K.V., Bastiaanssen T.F.S., Boehme M., Codagnone M.G., Cussotto S., Fulling C., Golubeva A.V. (2019). The Microbiota-Gut-Brain Axis. Physiol. Rev..

[15] Ji H.-F., Shen L. (2021). Probiotics as potential therapeutic options for Alzheimer’s disease. Appl. Microbiol. Biotechnol..

[16] Kowalski K., Mulak A. (2019). Brain-Gut-Microbiota Axis in Alzheimer’s Disease. J. Neurogastroenterol. Motil..

[17] Pluta R., Ułamek-Kozioł M., Januszewski S., Czuczwar S.J. (2020). Gut microbiota and pro/prebiotics in Alzheimer’s disease. Aging.

[18] Naomi R., Embong H., Othman F., Ghazi H.F., Maruthey N., Bahari H. (2021). Probiotics for Alzheimer’s Disease: A Systematic Review. Nutrients.

[19] Zhu G., Zhao J., Zhang H., Chen W., Wang G. (2021). Probiotics for Mild Cognitive Impairment and Alzheimer’s Disease: A Systematic Review and Meta-Analysis. Foods.

[20] Leng F., Edison P. (2021). Neuroinflammation and microglial activation in Alzheimer disease: Where do we go from here?. Nat. Rev. Neurol..

[21] Calsolaro V., Edison P. (2016). Neuroinflammation in Alzheimer’s disease: Current evidence and future directions. Alzheimer’s Dement..

[22] Schwab C., Klegeris A., McGeer P.L. (2010). Inflammation in transgenic mouse models of neurodegenerative disorders. Biochim. Biophys. Acta (BBA)—Mol. Basis Dis..

[23] Lin L., Zheng L.J., Zhang L.J. (2018). Neuroinflammation, Gut Microbiome, and Alzheimer’s Disease. Mol. Neurobiol..

[24] Giau V.V., Wu S.Y., Jamerlan A., An S.S.A., Kim S.Y., Hulme J. (2018). Gut Microbiota and Their Neuroinflammatory Implications in Alzheimer’s Disease. Nutrients.

[25] Mollazadeh H., Cicero A.F.G., Blesso C.N., Pirro M., Majeed M., Sahebkar A. (2019). Immune modulation by curcumin: The role of interleukin-10. Crit. Rev. Food Sci. Nutr..

[26] Porro C., Cianciulli A., Panaro M.A. (2020). The Regulatory Role of IL-10 in Neurodegenerative Diseases. Biomolecules.

[27] Su F., Bai F., Zhang Z. (2016). Inflammatory Cytokines and Alzheimer’s Disease: A Review from the Perspective of Genetic Polymorphisms. Neurosci. Bull..

[28] Toshimitsu T., Mochizuki J., Ikegami S., Itou H. (2016). Identification of a Lactobacillus plantarum strain that ameliorates chronic inflammation and metabolic disorders in obese and type 2 diabetic mice. J. Dairy Sci..

[29] Toshimitsu T., Ozaki S., Mochizuki J., Furuichi K., Asami Y. (2017). Effects of Lactobacillus plantarum Strain OLL2712 Culture Conditions on the Anti-inflammatory Activities for Murine Immune Cells and Obese and Type 2 Diabetic Mice. Appl. Environ. Microbiol..

[30] Toshimitsu T., Gotou A., Sashihara T., Hachimura S., Shioya N., Suzuki S., Asami Y. (2020). Effects of 12-Week Ingestion of Yogurt Containing Lactobacillus plantarum OLL2712 on Glucose Metabolism and Chronic Inflammation in Prediabetic Adults: A Randomized Placebo-Controlled Trial. Nutrients.

[31] Toshimitsu T., Gotou A., Sashihara T., Furuichi K., Hachimura S., Shioya N., Suzuki S., Asami Y. (2021). Ingesting Yogurt Containing *Lactobacillus plantarum* OLL2712 Reduces Abdominal Fat Accumulation and Chronic Inflammation in Overweight Adults in a Randomized Placebo-Controlled Trial. Curr. Dev. Nutr..

[32] Cho A., Sugimura M., Nakano S., Yamada T. (2008). The Japanese MCI Screen for Early Detection of Alzheimer’s Disease and Related Disorders. Am. J. Alzheimer’s Dis. Other Dement..

[33] Gualtieri C.T., Johnson L.G. (2006). Reliability and validity of a computerized neurocognitive test battery, CNS Vital Signs. Arch. Clin. Neuropsychol..

[34] Shankle W.R., Mangrola T., Chan T., Hara J. (2009). Development and validation of the Memory Performance Index: Reducing measurement error in recall tests. Alzheimer’s Dement..

[35] Toshimitsu T., Gotou A., Furuichi K., Hachimura S., Asami Y. (2019). Effects of 12-wk Lactobacillus plantarum OLL2712 treatment on glucose metabolism and chronic inflammation in prediabetic individuals: A single-arm pilot study. Nutrition.

[36] Cade J., Thompson R., Burley V., Warm D. (2002). Development, validation and utilisation of food-frequency questionnaires—A review. Public Health Nutr..

[37] Kobayashi S., Honda S., Murakami K., Sasaki S., Okubo H., Hirota N., Notsu A., Fukui M., Date C. (2012). Both Comprehensive and Brief Self-Administered Diet History Questionnaires Satisfactorily Rank Nutrient Intakes in Japanese Adults. J. Epidemiol..

[38] Kobayashi S., Murakami K., Sasaki S., Okubo H., Hirota N., Notsu A., Fukui M., Date C. (2011). Comparison of relative validity of food group intakes estimated by comprehensive and brief-type self-administered diet history questionnaires against 16 d dietary records in Japanese adults. Public Health Nutr..

[39] Sakurai K., Li H., Inamura N., Masuoka N., Hisatsune T. (2020). Relationship between elevated impulsivity and cognitive declines in elderly community-dwelling individuals. Sci. Rep..

[40] Momtaz Y.A., Haron S.A., Hamid T.A., Ibrahim R., Tanjani P.T. (2018). Body Mass Index (BMI) and Cognitive Functions in Later Life. Curr. Alzheimer Res..

[41] Sakurai K., Shen C., Shiraishi I., Inamura N., Hisatsune T. (2021). Consumption of Oleic Acid on the Preservation of Cognitive Functions in Japanese Elderly Individuals. Nutrients.

[42] Hair J.F., Anderson R.E., Tatham R.L., Black W.C. (1995). Multivariate Data Analysis.

[43] Seo E.H., Kim H., Choi K.Y., Lee K.H., Choo I.H. (2018). Pre-Mild Cognitive Impairment: Can Visual Memory Predict Who Rapidly Convert to Mild Cognitive Impairment?. Psychiatry Investig..

[44] Wang C.-S., Li W.-B., Wang H.-Y., Ma Y.-M., Zhao X.-H., Yang H., Qian J.-M., Li J.-N. (2018). VSL#3 can prevent ulcerative colitis-associated carcinogenesis in mice. World J. Gastroenterol..

[45] Wang C., Li W., Wang H., Ma Y., Zhao X., Zhang X., Yang H., Qian J., Li J. (2019). Saccharomyces boulardii alleviates ulcerative colitis carcinogenesis in mice by reducing TNF-α and IL-6 levels and functions and by rebalancing intestinal microbiota. BMC Microbiol..

[46] Jo J.-K., Seo S.-H., Park S.-E., Kim H.-W., Kim E.-J., Kim J.-S., Pyo J.-Y., Cho K.-M., Kwon S.-J., Park D.-H. (2021). Gut Microbiome and Metabolome Profiles Associated with High-Fat Diet in Mice. Metabolites.

[47] Zhang Y., Chen L., Hu M., Kim J.J., Lin R., Xu J., Fan L., Qi Y., Wang L., Liu W. (2020). Dietary type 2 resistant starch improves systemic inflammation and intestinal permeability by modulating microbiota and metabolites in aged mice on high-fat diet. Aging.

[48] Sydenham T.V., Arpi M., Klein K., Justesen U.S. (2014). Four Cases of Bacteremia Caused by Oscillibacter ruminantium, a Newly Described Species. J. Clin. Microbiol..

[49] Chen M., Hui S., Lang H., Zhou M., Zhang Y., Kang C., Zeng X., Zhang Q., Yi L., Mi M. (2019). SIRT3 Deficiency Promotes High-Fat Diet-Induced Nonalcoholic Fatty Liver Disease in Correlation with Impaired Intestinal Permeability through Gut Microbial Dysbiosis. Mol. Nutr. Food Res..

[50] Kim C.C., Healey G.R., Kelly W.J., Patchett M.L., Jordens Z., Tannock G.W., Sims I.M., Bell T.J., Hedderley D., Henrissat B. (2019). Genomic insights from Monoglobus pectinilyticus: A pectin-degrading specialist bacterium in the human colon. ISME J..

[51] Chen Z., Wu S., Zeng Y., Chen Z., Li X., Li J., He L., Chen M. (2022). FuZhengHuaYuJiangZhuTongLuoFang Prescription Modulates Gut Microbiota and Gut-Derived Metabolites in UUO Rats. Front. Cell Infect. Microbiol..

[52] Vaziri N.D., Yuan J., Norris K. (2013). Role of Urea in Intestinal Barrier Dysfunction and Disruption of Epithelial Tight Junction in Chronic Kidney Disease. Am. J. Nephrol..

[53] Ip W.K.E., Hoshi N., Shouval D.S., Snapper S., Medzhitov R. (2017). Anti-inflammatory effect of IL-10 mediated by metabolic reprogramming of macrophages. Science.

[54] Quiros M., Nishio H., Neumann P.A., Siuda D., Brazil J.C., Azcutia V., Hilgarth R., O’Leary M.N., Garcia-Hernandez V., Leoni G. (2017). Macrophage-derived IL-10 mediates mucosal repair by epithelial WISP-1 signaling. J. Clin. Investig..

[55] Wang Y., Yu P., Li Y., Zhao Z., Wu X., Zhang L., Feng J., Hong J.-S. (2021). Early-Released Interleukin-10 Significantly Inhibits Lipopolysaccharide-Elicited Neuroinflammation In Vitro. Cells.

[56] Ozben T., Ozben S. (2019). Neuro-inflammation and anti-inflammatory treatment options for Alzheimer’s disease. Clin. Biochem..

[57] Kiyota T., Ingraham K.L., Swan R.J., Jacobsen M.T., Andrews S.J., Ikezu T. (2012). AAV serotype 2/1-mediated gene delivery of anti-inflammatory interleukin-10 enhances neurogenesis and cognitive function in APP+PS1 mice. Gene Ther..

[58] Takano T., Endo R., Wang Y., Nakajima-Adachi H., Hachimura S. (2020). Lactobacillus plantarum OLL2712 induces IL-10 production by intestinal dendritic cells. Biosci. Microbiota Food Health.

[59] Ooi L.-G., Liong M.-T. (2010). Cholesterol-Lowering Effects of Probiotics and Prebiotics: A Review of in Vivo and in Vitro Findings. Int. J. Mol. Sci..

[60] Liu C.-C., Kanekiyo T., Xu H., Bu G. (2013). Apolipoprotein E and Alzheimer disease: Risk, mechanisms and therapy. Nat. Rev. Neurol..

[61] Anstey K.J., Ashby-Mitchell K., Peters R. (2017). Updating the Evidence on the Association between Serum Cholesterol and Risk of Late-Life Dementia: Review and Meta-Analysis. J. Alzheimer’s Dis..

[62] Berger S., Raman G., Vishwanathan R., Jacques P.F., Johnson E.J. (2015). Dietary cholesterol and cardiovascular disease: A systematic review and meta-analysis. Am. J. Clin. Nutr..

[63] Grimm M.O.W., Mett J., Hartmann T. (2016). The Impact of Vitamin E and Other Fat-Soluble Vitamins on Alzheimer’s Disease. Int. J. Mol. Sci..

[64] Alisi L., Cao R., De Angelis C., Cafolla A., Caramia F., Cartocci G., Librando A., Fiorelli M. (2019). The Relationships Between Vitamin K and Cognition: A Review of Current Evidence. Front. Neurol..

[65] Chouet J., Ferland G., Feart C., Rolland Y., Presse N., Boucher K., Barberger-Gateau P., Beauchet O., Annweiler C. (2015). Dietary Vitamin K Intake Is Associated with Cognition and Behaviour among Geriatric Patients: The CLIP Study. Nutrients.

[66] Soutif-Veillon A., Ferland G., Rolland Y., Presse N., Boucher K., Féart C., Annweiler C. (2016). Increased dietary vitamin K intake is associated with less severe subjective memory complaint among older adults. Maturitas.

[67] Presse N., Shatenstein B., Kergoat M.J., Ferland G. (2008). Low vitamin K intakes in community-dwelling elders at an early stage of Alzheimer’s disease. J. Am. Diet Assoc..

[68] Ferland G., Feart C., Presse N., Lorrain S., Bazin F., Helmer C., Berr C., Annweiler C., Rouaud O., Dartigues J.-F. (2016). Vitamin K Antagonists and Cognitive Function in Older Adults: The Three-City Cohort Study. J. Gerontol. Ser. A.

[69] Martin-Gallausiaux C., Marinelli L., Blottiere H.M., Larraufie P., Lapaque N. (2021). SCFA: Mechanisms and functional importance in the gut. Proc. Nutr. Soc..

[70] Gallardo G., Holtzman D.M. (2019). Amyloid-β and Tau at the Crossroads of Alzheimer’s Disease. Adv. Exp. Med. Biol..

