# Supplementary material for: Effects of Lactiplantibacillus plantarum OLL2712 on Memory Function in Older Adults with Declining Memory: A Randomized Placebo-Controlled Trial
Source: Nutrients. 2022 Oct 14;14(20):4300. doi: 10.3390/nu14204300 (PMC9610166; doi:10.3390/nu14204300)
Supplement: Supplementary file 1 [file nutrients-14-04300-s001.zip › nutrients-1938289-supplementary/Supplementary Table S2.pdf]

Table S2. Measures of visual, verbal, and composite memory in Cognitrax

Test Name	Example of screen	Test Procedure	Score Calculation
Visual Memory (VIM)	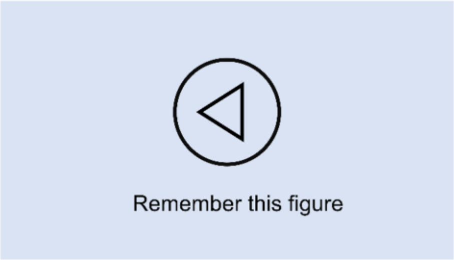	VIM measures memory function for words. First, 15 figures are presented, one every 2 seconds, and the subject memorizes them. After that, 30 figures, including new figures, are presented, one every 2 seconds, and the subject identifies the figure that he/she memorized. Then, after 25 minutes, there is a delayed recognition trial.	Number of correct hits for immediate recognition + Number of correct passes for immediate recognition + Number of correct hits for delayed recognition + Number of correct passes for delayed recognition
Verbal Memory (VBM)	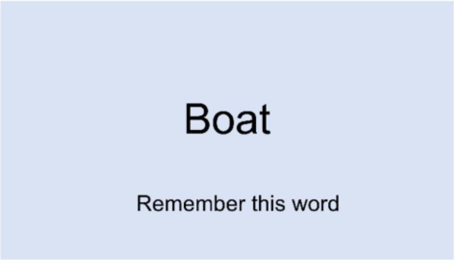	VBM measures memory function for words. First, 15 words are presented, one every 2 seconds, and the subject memorizes them. After that, 30 words, including new words, are presented, one every 2 seconds, and the subject identifies the word that he/she memorized. Then, after 25 minutes, there is a delayed recognition trial.	Number of correct hits for immediate recognition + Number of correct passes for immediate recognition + Number of correct hits for delayed recognition + Number of correct passes for delayed recognition
Composite Memory			VIM score + VBM score
